# Synthesis and utility of *N*-boryl and *N*-silyl enamines derived from the hydroboration and hydrosilylation of *N*-heteroarenes and *N*-conjugated compounds

**DOI:** 10.3389/fchem.2024.1414328

**Published:** 2024-06-07

**Authors:** Vinh Do Cao, Seewon Joung

**Affiliations:** Department of Chemistry, Inha University, Incheon, Republic of Korea

**Keywords:** hydroboration, hydrosilylation, *N*-boryl enamine, *N*-silyl enamine, *N*-heteroarene, unsaturated nitriles, aldimines

## Abstract

Catalytic hydroboration and hydrosilylation have emerged as promising strategies for the reduction of unsaturated hydrocarbons and carbonyl compounds, as well as for the dearomatization of *N*-heteroarenes. Various catalysts have been employed in these processes to achieve the formation of reduced products via distinct reaction pathways and intermediates. Among these intermediates, *N*-silyl enamines and *N*-boryl enamines, which are derived from hydrosilylation and hydroboration, are commonly underestimated in this reduction process. Because these versatile intermediates have recently been utilized *in situ* as nucleophilic reagents or dipolarophiles for the synthesis of diverse molecules, an expeditious review of the synthesis and utilization of *N*-silyl and *N*-boryl enamines is crucial. In this review, we comprehensively discuss a wide range of hydrosilylation and hydroboration catalysts used for the synthesis of *N*-silyl and *N*-boryl enamines. These catalysts include main-group metals (e.g., Mg and Zn), transition metals (e.g., Rh, Ru, and Ir), earth-abundant metals (e.g., Fe, Co, and Ni), and non-metal catalysts (including P, B, and organocatalysts). Furthermore, we highlight recent research efforts that have leveraged these versatile intermediates for the synthesis of intriguing molecules, offering insights into future directions for these invaluable building blocks.

## 1 Introduction

**FIGURE 1:**
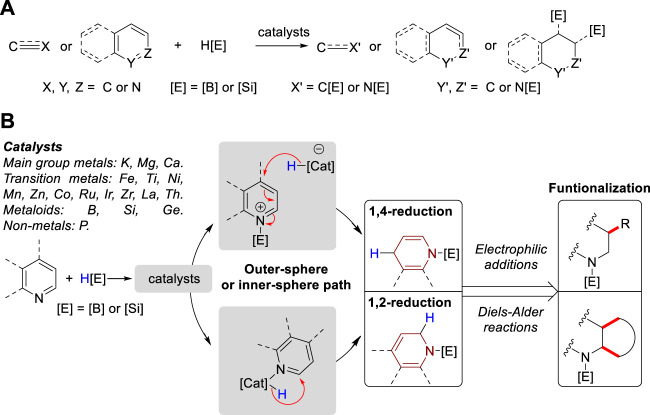
**(A)** Previous reviews on hydroelementation of pi-bond system. **(B)** This review: Syntheses of *N*-boryl and *N*-silyl enamines using hydroboration and hydrosilylation.

Enamine is a functional moiety found in a variety of natural products, bioactive molecules, and pharmaceuticals (Burgess et al., 1991; Goldmann and Stoltefuss, 1991; Gordeev et al., 1998; Edafiogho et al., 2007; Sashidhara et al., 2009; Poulsen, 2021). Within the realm of synthetic organic chemistry, enamines serve as versatile intermediates (Fu et al., 2019; Wang et al., 2023) and catalysts (Notz et al., 2004; Mukherjee et al., 2007; Zou et al., 2018) to facilitate the synthesis of diverse chemical structures, including natural products and heterocyclic compounds (Cheng et al., 2004; Hanessian and Chattopadhyay, 2014; Wang, 2015). Traditionally, enamines are prepared through condensation reactions between secondary amines and carbonyl compounds, α,β-elimination of amides, or reductive acylation of ketoximes (Mengya, 2018). However, these conventional methods give low conversion or exhibit narrow functional group tolerance due to their harsh reaction conditions (Dehli et al., 2005). Consequently, numerous alternative methods have been developed, including the amination of alkenes or alkynes (Beller et al., 2002; Ahmed et al., 2003), methylenation of amides (Shen and Porco, 2000), dehydrogenation of tertiary amines (Zhang et al., 2003), cross-coupling of amines and alkenyl bromides (Barluenga et al., 2004), and *N*-formylation of amines with CO_2_ and PhSiH_3_ to synthesize linear enamines (Nakaoka et al., 2023). Furthermore, the cobalt-catalyzed hydrogen transfer of amines (Bolig and Brookhart, 2007) and palladium-catalyzed intramolecular amination of alkenes (Jiang et al., 2017) are utilized to synthesize cyclic enamines. In addition, the hydroboration and hydrosilylation of *N*-heteroarenes and conjugated imine or nitrile compounds are also used to obtain enamine derivatives, particularly borylated and silylated enamines. In contrast to traditional reductive methods such as metal hydride reduction and hydrogenation, the use of silanes and boranes as reducing agents enables milder reaction conditions, high chemo- and regioselectivity, and compatibility with diverse functional groups (Park and Chang, 2017). Consequently, hydroboration and hydrosilylation have emerged as alternatives to the use of H_2_, which is a common method in catalytic reduction chemistry (Marciniec, 2005; Arai and Ohkuma, 2008). Moreover, hydroboration and hydrosilylation demonstrate catalytic versatility, as they can be facilitated by various metal-based complexes, metalloids, and non-metal compounds. The initial steps, depending on the activity of the catalyst, may involve the activation of the B–H bond in HBpin and the Si–H bond in silanes, or coordination with heteroatom centers in the reactant substrates. However, the general catalytic mechanisms can be categorized into outer-sphere and inner-sphere pathways (Park and Brookhart, 2010; Iglesias et al., 2014; Corey, 2016; Lipke et al., 2017), which determine the hydride attack pathway through the formation of distinct intermediates. The chemical reactivities, selectivities, and primary mechanistic insights of these hydroboration and hydrosilylation reactions have been extensively discussed in reviews focusing on the hydroelementation of alkene (Du and Huang, 2017), alkyne (Saptal et al., 2020), nitrile (Das et al., 2022), as well as dearomatization of unactivated *N*-heteroarenes (Park and Chang, 2017; Park, 2020; Escolano et al., 2024). A borane-catalyzed double hydrosilylation for the formation of sp^3^ C–Si bonds (Park, 2019) and single hydroelementation reaction of *N*-heteroarenes were also reviewed (Park, 2024) (Figure 1A). However, a review focusing on the synthesis and utility of *N*-boryl and *N*-silyl enamines has not yet been reviewed. Thus, in this review, we first outline the formation of *N*-boryl and *N*-silyl enamines via the 1,4-reduction of quinolines, conjugated nitriles/aldimines, 1,2-reduction of isoquinoline, and both 1,2- and 1,4-reduction of pyridines. Then, we summarized their subsequent applications in nucleophilic additions and Diels–Alder reactions (Figure 1B).

## 2 Hydroboration in the synthesis of *N*-boryl enamines

### 2.1 Alkali- and alkaline-earth-metal-catalyzed hydroboration of *N*-heteroarenes

**FIGURE 2:**
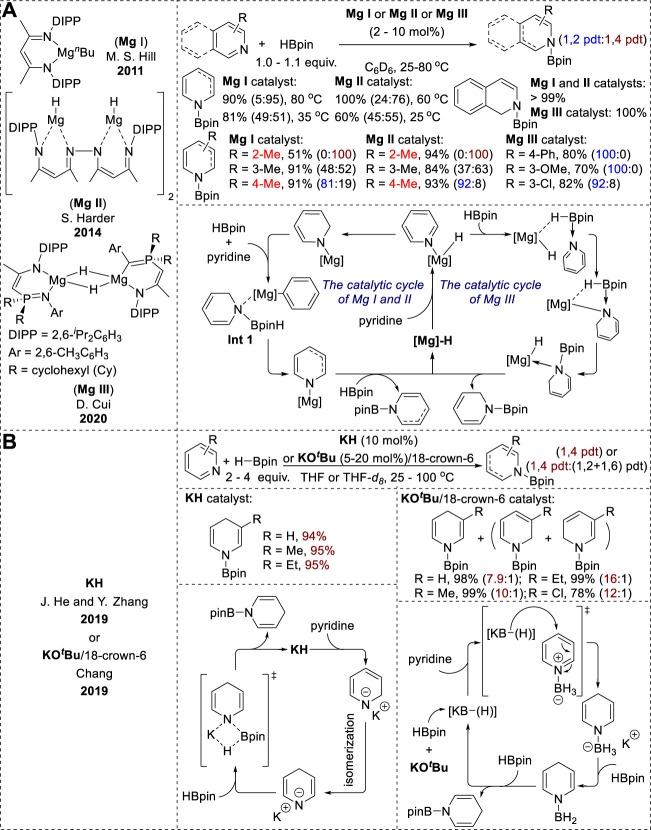
**(A)** Alkaline-earth-metal-catalyzed hydroboration of *N*-heteroarenes. **(B)** Alkali-metal-catalyzed hydroboration of *N*-heteroarenes.

The magnesium-catalyzed hydroboration of pyridines and isoquinolines has evolved from mononuclear structures (**Mg I**) to the dinuclear β-diketiminate magnesium hydrides (**Mg II**). Recently, a phosphinimino-amido magnesium complex (**Mg III**) has also been reported (Figure 2A). While the **Mg I** and **Mg II** systems exhibited temperature-dependent selectivity between 1,2- and 1,4-hydroboration (Arrowsmith et al., 2011; Intemann et al., 2014), the **Mg III** catalyst showed highly selective 1,2-hydroboration (Liu et al., 2020). Furthermore, the formation of both regioisomers under **Mg I** and **Mg II** conditions indicated that the magnesium hydride complex was not a key step in these mechanisms. Instead, the generation of an intermediate (**Int 1**, Figure 2) through the reaction of the [Mg]-1,2-reduced intermediate with HBpin directly transfers a hydride from boron to the 2- or 4-position of the pyridine ligand (Arrowsmith et al., 2011; Intemann et al., 2014). Moreover, density functional theory (DFT) calculations using the **Mg III** catalytic system showed that the dearomatization process resulted in a reasonable energy barrier for the 1,2-reduced intermediate, whereas 1,4-dearomatization presented considerable challenges because of its high energy demand (Liu et al., 2020).

Recently, Zhang et al. and Chang et al. reported the potassium-catalyzed hydroboration of *N*-heteroarenes (Figure 2B). Both catalytic conditions resulted in the highly selective 1,4-hydroboration of pyridines (Liu et al., 2019; Jeong et al., 2019). However, the KH catalytic systems reported by Zhang exhibited excellent 1,2-selectivity with quinoline substrates. The use of THF solvents and elevated reaction temperatures enhanced the 1,4-selectivity in the hydroboration of pyridines. This selectivity is due to the formation of a 1,4-reduced intermediate through the thermodynamic isomerization of the 1,2-reduced intermediate (Liu et al., 2019). Conversely, the 1,4-regioselectivity in the KO^*t*^Bu-promoted hydroboration in Chang’s study originated from an outer-sphere mechanism. KO^*t*^Bu and HBpin react to form various borohydride species as active hydrides in the presence of BH_3_. These active hydride species then generate 1,4-reduced intermediates via nucleophilic hydride attack on the pyridine–BH_3_ adducts. Subsequent hydride transfer generates *N*-borylated-1,4-dihydropyridines, while liberating BH_3_ through σ-bond metathesis (Jeong et al., 2019).

### 2.2 Earth-abundant transition-metal-catalyzed 1,4-hydroboration

In addition to the main group of metals, various earth-abundant transition metal catalysts, such as Ni(acac)_2_/phosphine ligand, 1-Methylimidazole-based Mn pincer complex [LMn(CO)_2_], and the heterobinuclear Cu/Fe catalyst (IPr)CuFp exhibited 1,4-selective hydroboration of pyridines and quinolines (Figure 2). In the (IPr)CuFp catalytic system, 3-substituted pyridines with electron-donating groups underwent effective reduction with excellent yields, whereas 2- and 4-substituted pyridines exhibited minimal-to-no conversion (Yu et al., 2020). Conversely, Ni(acac)_2_/PCyp_3_ circumvented steric hindrance at the 4-position of pyridine. Furthermore, electron-rich substituents at the 3-position enhanced the reactivity, resulting in high yields, whereas electron-poor pyridines showed no reactivity (Tamang et al., 2018); (LMn(CO)_2_) exhibited 1,4-hydroboration reactivity on various substituted quinolines, resulting in full conversion with high regioselectivity (Wang et al., 2023).

**FIGURE 3:**
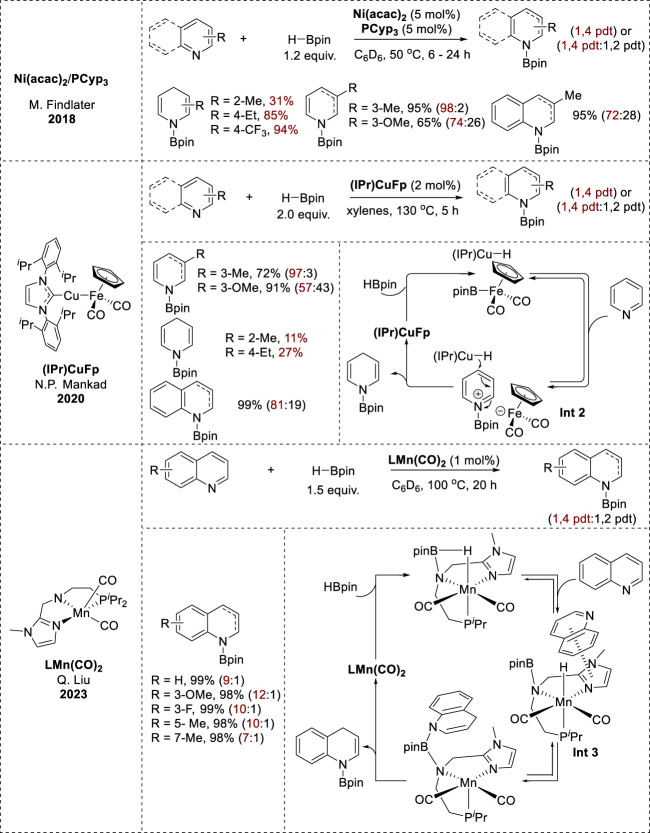
Earth-abundant transition-metal-catalyzed 1,4-hydroboration.

Kinetic and mechanistic investigations of the Ni(acac)_2_/PCyp_3_ catalytic system revealed the formation of bis(heteroarene) complexes as the first step (Tamang et al., 2018). In contrast, the (IPr)CuFp catalytic system formed (IPr)CuH as an active hydride species. Pyridine is then activated to form a pyridyl cation. *N*-borylated-1,4-dihydropyridines are produced through the interaction of an active hydride species at the 4-position of the pyridyl cation (**Int 2**, Figure 3) (Yu et al., 2020). Additionally, mechanistic studies of [LMn(CO)_2_] showed that the 1,2-adduct was kinetically favorable, whereas the 1,4-adduct was more stable and was generated via a thermodynamic process. The unusual 1,4-regioselectivity was derived from the decreased free-energy barrier for the 1,4-hydroboration compared to that for the 1,2-hydroboration. This could be achieved by using a 1-methylimidazole-based pincer Mn catalyst featuring cooperative C–H···N and π···π noncovalent interactions between the 1-methylimidazole moiety and the quinoline substrate (**Int 3**, Figure 3) (Wang et al., 2023).

### 2.3 Earth-abundant transition-metal-catalyzed 1,2-hydroboration

Recent studies have reported the 1,2-selective hydroboration of *N*-heteroarenes catalyzed by a variety of earth-abundant transition metals (Figure 3). It is generally assumed that the mechanistic pathways involve various hydride intermediate complexes, including metal hydrides and borohydrides, facilitating the hydride shift to the C_2_-position in *N*-heteroarenes.

The N_2_-bridged diiron complex [Cp*-(Ph_2_PC_6_H_4_S)Fe]_2_ (μ-N_2_), zinc alkyl complex LZnEt, Cp*Ni(1,2-Ph_2_PC_6_H_4_O), and Mn (hmds)_2_ demonstrated good reactivity with high 1,2-selectivity towards various substituted pyridines and isoquinolines (Zhang et al., 2017; Liu et al., 2019; Wang et al., 2020; Ghosh and Jacobi von Wangelin, 2021). Next, the zinc hydride NacNacZnH exhibited limited 1,2-hydroboration reactivity with a few pyridine substrates to give cyclic *N*-boryl enamines (Lortie et al., 2017). Additionally, a cobalt-based complex ((PPh_3_)_3_CoH(N_2_)/pincer) with an *N*-heterocyclic carbene ligand (^Me^CNC) showed poor regioselectivity depending on the position of the substituent in the pyridines. Specifically, pyridine derivatives with diverse functional groups at the 4-position afforded excellent yields of the 1,2-hydroborylated products, whereas unsubstituted pyridines and pyridines with C3 substituents showed a notable preference for 1,4-hydroboration products (Meher et al., 2023).

**FIGURE 4:**
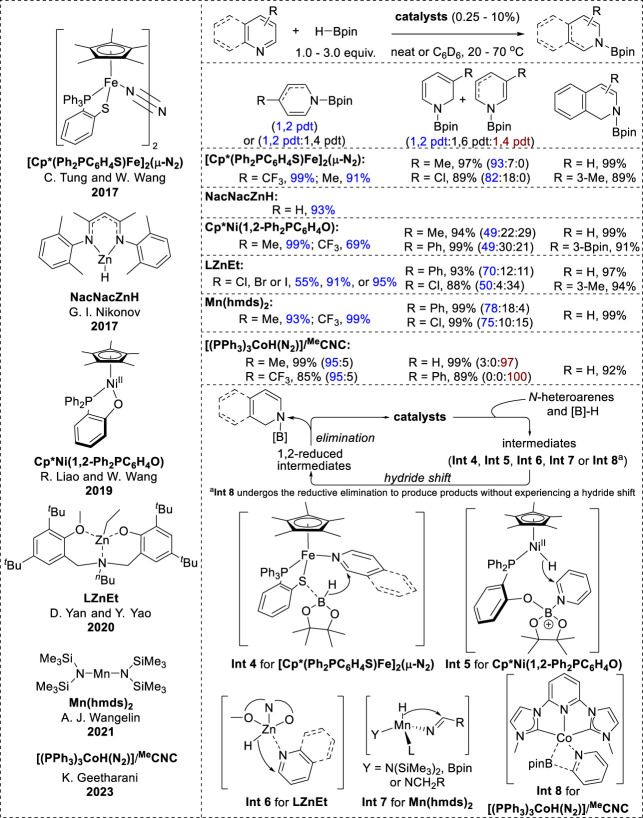
Earth-abundant transition-metal-catalyzed 1,2-hydroboration.

In accordance with the inner-sphere pathway, the hydroboration mechanism of the zinc alkyl complexes LZnEt and Mn (hmds)_2_ involves the formation of metal hydride species through the interaction of metal catalysts with HBpin in the initial step (Wang et al., 2020; Ghosh and Jacobi von Wangelin, 2021). The insertion of a hydride into C=N in *N*-heteroarenes (**Int 6**, **Int 7** in Figure 4) generates a 1,2-reduced intermediate complex that undergoes metathesis with HBpin to form *N-*boryl-dihydropyridines.

Differently from conventional catalytic mechanisms, (IPr)CuFp activates the B–H bond in HBpin, resulting in the generation of hydride species, whereas the iron–thiolate catalyst coordinates with *N*-heteroarenes and the thiolate interacts with HBpin. This interaction further facilitates hydride transfer from HBpin to the C2 position of the *N*-heteroarene ligand (**Int 4**, Figure 4) (Zhang et al., 2017). Additionally, the catalytic reaction of Cp*Ni(1,2-Ph_2_PC_6_H_4_O) is initiated by the dissociation of the B–H bond via the activation of HBpin and the nickel (II) complex, forming a nickel (II) hydride with an oxygen-stabilized boron moiety. Subsequently, nickel hydride is transferred to the C2-position of pyridine, which coordinates with the boron atom of the ligand (**Int 5**, Figure 4) (Liu et al., 2019). Eventually, *N*-borylated-1,2-dihydropyridine is generated through cleavage of the O–B bond. Similarly, the proposed catalytic cycle of (PPh_3_)_3_CoH(N_2_)/(^Me^CNC) starts with the reaction of the in-situ-formed cobalt hydride [(^Me^CNC)CoH] with HBpin. This reaction generates [(^Me^CNC)Co(Bpin)] and simultaneously liberates H_2_ without a hydride shift to C=N in pyridines. **Int 8** (Figure 4) was then formed, as [(^Me^CNC)Co(Bpin)] was coordinated with pyridine. Subsequent transfer of the Bpin moiety, followed by the reductive elimination of **Int 8**, resulted in the formation of the *N*-boryl enamine product (Meher et al., 2023).

### 2.4 Metalloid and non-metal catalyzed 1,4-hydroboration

**FIGURE 5:**
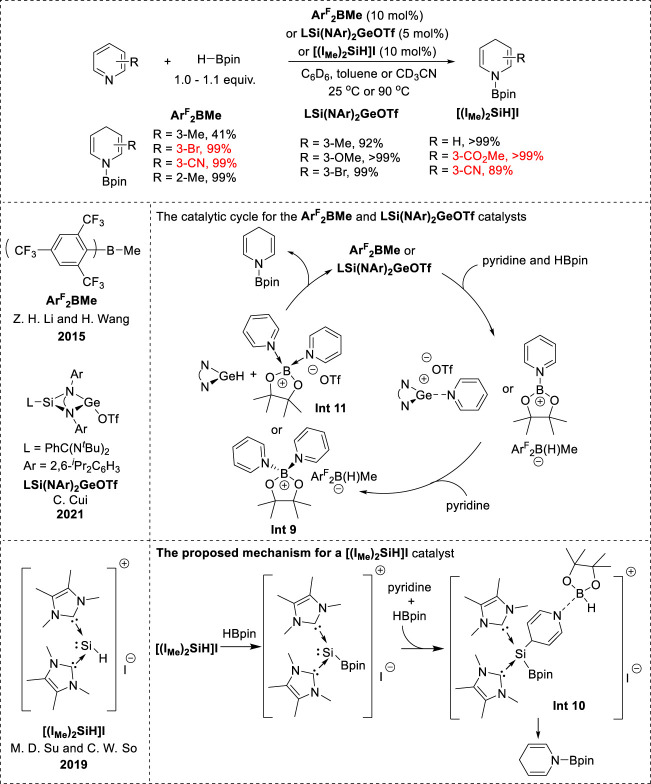
Metalloid-catalyzed 1,4-hydroboration.

Recently, the borane Ar^F^_2_BMe, NHC-parent silyliumylidene cation complex [(IMe)_2_SiH]I, and *N*-heterocyclic germylene LSi(NAr)_2_GeOTf (L = PhC(N^*t*^Bu)_2_ and Ar = 2,6-^*i*^Pr_2_C_6_H_3_) were utilized for the metalloid-catalyzed hydroboration of pyridines (Figure 4). These catalysts demonstrated highly selective 1,4-hydroboration of various substituted pyridines. Notably, in the borane (Ar^F^_2_BMe) catalytic system, 3-substituted pyridines with electron-donating groups decreased the reactivity and electron-withdrawing groups accelerated the reactivity with excellent chemoselectivity (Fan et al., 2015). Pyridines bearing C=O and CN groups were tolerated under [(IMe)_2_SiH]I catalyst conditions (Leong et al., 2019). Moreover, LSi(NAr)_2_GeOTf exhibited notable efficiency in the 1,4-hydroboration of 3-substituted pyridines bearing both electron-donating and electron-withdrawing groups **(** Hu et al., 2021). In the Ar^F^_2_BMe and LSi(NAr)_2_GeOTf catalytic systems, the mechanism involved an ionic hydroboration. The formation of intermediate **Int 9** (Figure 5) and the [ArF_2_B(H)Me] anion hydride, as well as that of **Int 11** (Figure 5) and the germylene hydride, plays a crucial role. 1,4-Reduction occurs via hydride transfer from the [Ar^F^_2_B(H)Me] anion or germylene hydride to the 4-position of the pyridine in **Int 9** or **Int 11**, resulting in the formation of *N*-boryl 1,4-dihydropyridine. Notably, the steric effects of the silaamidinate ligand or bulky borohydride may determine the 1,4-selectivity when the hydride attacks the pyridine moiety (Fan et al., 2015; Hu et al., 2021). In contrast, the catalytic mechanism of [(IMe)_2_SiH]I is initiated by the HBpin-activating silylium catalyst, which facilitates a nucleophilic attack at the C4-position of the pyridines to form the hydroborated intermediate **Int 10** (Figure 5). Subsequently, **Int 10** underwent hydride transfer from the borane moiety to the C4-position, replacing the C–Si bond and leading to the regeneration of the catalyst and formation of *N*-boryl-1,4-dihydropyridines (Leong et al., 2019).

**FIGURE 6:**
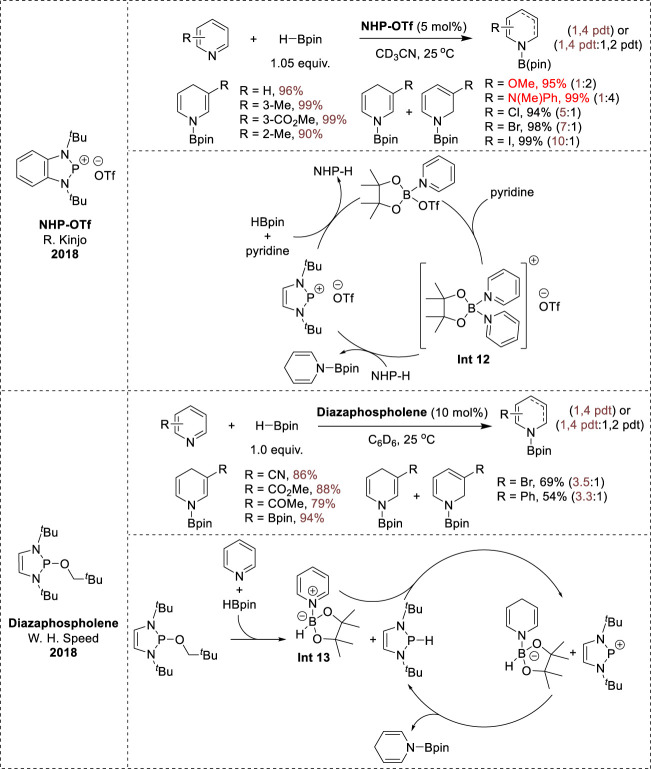
Non-metal-catalyzed 1,4-hydroboration.

In 2018, two distinct non-metal catalytic systems were reported for the 1,4-hydroboration of pyridine. Chong and Kinjo’s group utilized *N*-heterocyclic phosphenium triflates (NHP-OTf) as catalysts and demonstrated their regioselectivity and tolerance to electron-donating groups (Rao et al., 2018). In contrast, Speed et al. employed a neutral diazaphospholene catalyst that exhibited superior selectivity towards certain electron-deficient pyridines, and the reaction operated well in solvents with low polarity, such as benzene-d_6_ or diethyl ether, in which Kinjo’s procedure failed (Hynes et al., 2018). The reactions with both cationic and neutral phosphor catalysts resulted in high yields and chemoselectivities. The reaction mechanism for phosphenium triflates (NHP-OTf) was initiated by activation of the B–H bond of HBpin with pyridine and a phosphonium triflate catalyst, leading to the formation of the Py-Bpin-OTf complex. This intermediate complex then coordinates with a second pyridine molecule to afford the boronium [(Py)_2_∙Bpin]OTf (**Int 12**, Figure 6). Subsequently, one of the activated pyridine moieties in **Int 12** undergoes reduction via hydride transfer from NHP-H, resulting in the formation of either *N*-boryl 1,2-dihydropyridine or *N*-boryl 1,4-dihydropyridine, while simultaneously regenerating the phosphonium catalyst (Rao et al., 2018). However, the neutral diazaphospholene catalyst facilitated the interaction of pyridine with H-Bpin, resulting in the formation of a pyridinium borate complex (**Int 13**, Figure 6), which was not formed during Kinjo’s catalytic cycle, along with diazaphospholene hydride. Subsequently, this **Int 13** complex undergoes hydride transfer from the diazaphospholene hydride to the 4-position of the pyridine moiety, yielding a dihydropyridyl borate complex paired with a phosphenium cation. Eventually, the transfer of the hydride from the borate complex to the phosphonium cation regenerates the diazaphospholene hydride and releases *N-*borylated-1,4-dihydropyridine (Hynes et al., 2018).

### 2.5 Noble d-block transition-metal-catalyzed-hydroboration

AgSbF_6_ has proven to be an effective catalyst for the hydroboration of various unsaturated functionalities, including isocyanates, pyridines, and quinolines (Pandey et al., 2022). However, only pyridine substrates gave *N*-boryl enamines via 1,4-hydroboration. Good-to-excellent yields of the 1,4-hydroborated products were obtained from 3-substituted pyridines. In addition, the hydroboration of 3,5-disubstituted pyridines was more efficient for electron-withdrawing than electron-donating substituents, but 2-substituted pyridines failed to undergo hydroboration. In contrast to previous reports, control experiments revealed that silver-salt-catalyzed hydroboration is a radical-mediated process.

**FIGURE 7:**
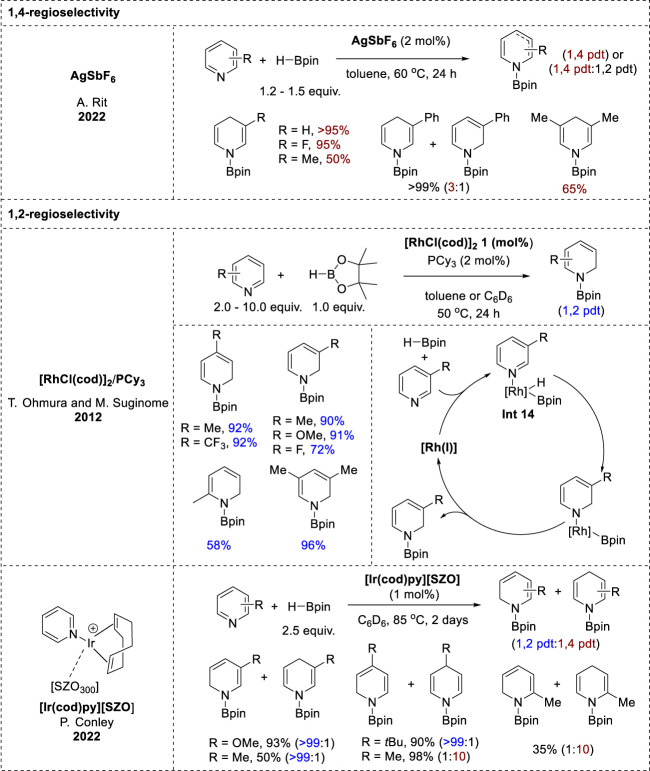
Noble d-block transition-metal-catalyzed-hydroboration.

The 1,2-selective hydroboration of pyridines using noble d-block transition-metal catalysts involved two distinct catalytic systems (Figure 7). One system utilized the iridium catalyst [Ir (cod)py][SZO], which was synthesized from (cod)IrCl(py) and [Me_3_Si][SZO_300_] as part of Conley’s research (Rodriguez and Conley, 2022), whereas the other employed the rhodium catalyst [RhCl(cod)]_2_ developed by Ohmura and Suginome’s group (Oshima et al., 2012). A comparison of the reactivities of pyridines with the Rh and Ir catalysts revealed notable differences. With the rhodium catalyst, various pyridines exhibited high-to-moderate yields of the corresponding *N*-borylated 1,2-dihydropyridines with good selectivity (Oshima et al., 2012), while with an iridium catalyst, the pyridines tended to yield mixtures of the 1,2- and 1,4-hydroboration products, or showed a preference for one of the products based on the substituent pattern (Rodriguez and Conley, 2022). In the rhodium-catalyzed hydroboration, the key intermediate is **Int 14** (Figure 7), which was formed through the oxidative addition of the B–H bond of HBpin to Rh(I), along with pyridine coordination. Pyridine insertion into the Rh–H bond at the 1,2-positions led to the formation of boryl rhodium amide, and the subsequent reductive elimination yielded the *N*-boryl enamine and regenerated Rh(I) (Oshima et al., 2012). Further research is needed to understand the mechanistic details of the hydroboration catalyzed by [Ir (cod)py][SZO], which likely proceeds via an inner-sphere pathway involving an iridium boryl hydride intermediate or the interaction of the pyridyl nitrogen with the Ir-BPin species (Rodriguez and Conley, 2022).

### 2.6 f-block transition-metal-catalyzed-hydroboration

**FIGURE 8:**
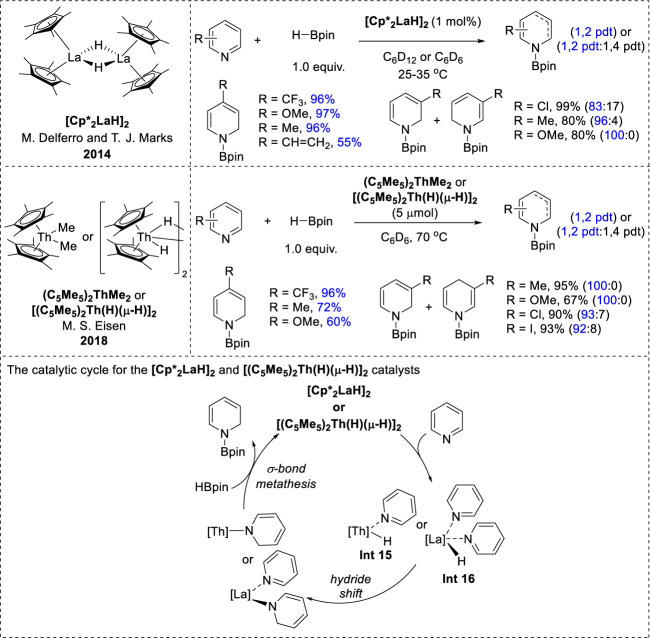
f-Block transition-metal-catalyzed hydroboration.

Efficient f-block transition-metal catalysts have recently emerged for the 1,2-regioselective hydroboration of pyridines. Notable examples include organolanthanide [Cp*_2_LaH]_2_ and thorium complexes such as thorium methyl (C_5_Me_5_)_2_ThMe_2_ and thorium hydride [(C_5_Me_5_)_2_Th(H) (μ-H)]_2_ complexes (Figure 8). These catalysts demonstrated similar reactivity profiles and high 1,2-regioselectivity for pyridine substrates. Specifically, ortho-substituted pyridines exhibited negligible activity under both catalytic conditions because of steric hindrance at the 2-position, whereas meta- and para-functionalized pyridines yielded *N*-boryl dihydropyridines in good yields and excellent selectivities. However, it was observed that the conjugated substituents could not be tolerated under thorium catalysis conditions (Dudnik et al., 2014; Liu et al., 2018). Furthermore, the catalytic mechanism underlying these transformations closely resembles that of other metal-catalyzed hydroboration reactions. It proceeds via an inner-sphere pathway, with the metal hydride species playing a pivotal role in the 1,2-addition of La–H or Th–H to the C=N bond of the coordinated pyridine, leading to the formation of a dihydropyridine complex intermediate. Subsequently, these intermediates undergo σ-bond metathesis with another HBPin molecule to afford *N*-borylated dihydropyridines (Dudnik et al., 2014; Liu et al., 2018).

## 3 Hydrosilylation in synthesis of *N*-silyl enamines

### 3.1 Earth-abundant-metal-catalyzed 1,2-hydrosilylation of *N*-heteroarenes

**FIGURE 9:**
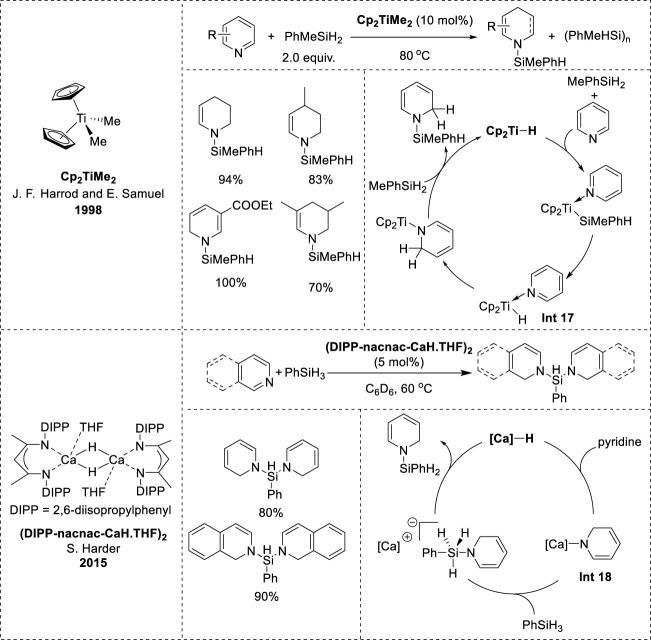
Earth-abundant-metal-catalyzed 1,2-hydrosilylation of *N*-heteroarenes.

Harrod and Samuel’s research on titanocene-catalyzed hydrosilylation began in 1998 and was further explored in a subsequent report in 2001. Their results revealed that Cp_2_TiMe_2_ effectively promoted the hydrosilylation of pyridine, yielding high amounts of *N*-silyl-tetrahydropyridine with MePhSiH_2_, but was ineffective for hydrosilylation with PhSiH_3_ or Ph_2_SiH_2_ (Hao et al., 1998). In contrast, changing the ligand to Cp*_2_TiMe_2_ led to successful hydrosilylation with PhSiH_3_, whereas using PhMeSiH_2_ instead of PhSiH_3_ resulted in a significantly slower reaction rate than that with Cp_2_TiMe_2_ (Harrod et al., 2001). The mechanism of titanocene-catalyzed hydrosilylation involves the interaction of Cp_2_TiMe_2_ with silane and pyridine to form a hydride complex (**Int 17**, Figure 9). The formation of **Int 17** was not observed directly in the reaction, but was supported by model stoichiometric reactions. Moreover, the hydride complex **Int 17** is believed to be the key intermediate in the dearomatization of pyridine, followed by the insertion of the Ti–H bond into the N=C bond of pyridine to form a 1,2-dihydropyridine complex (Hao et al., 1998; Harrod et al., 2001). Eventually, the 1,2-dihydropyridine complex underwent σ-bond metathesis with silane to produce the *N*-silyl-1,2-dihydropyridine product and regenerate Cp_2_TiH.

In 2015, Harder et al. embarked on an investigation into the reactivity of the calcium hydride complex (DIPP-nacnac-CaH∙THF)_2_ ([Ca]–H) based on their prior research on hydroboration catalyzed by magnesium hydride complexes (Intemann et al., 2015). Their initial observations revealed that [Ca]–H exhibited higher reactivity and 1,2-reduction selectivity in the dearomatization of pyridine than the magnesium hydride complex, with 1,2 to 1,4 isomerization upon increasing the temperature (Arrowsmith et al., 2011; Intemann et al., 2014). The formation of the calcium 1,2-DHP complex revealed the catalytic potential of [Ca]–H for hydroboration and hydrosilylation. However, unlike magnesium hydride, [Ca]–H was inactive in the hydroboration of pyridine owing to the predominant formation of B_2_ (pin)_3_ rather than the desired hydroboration product, although it proved to be efficient in hydrosilylation. Pyridines and isoquinolines were effectively reduced to afford excellent product yields. Furthermore, the mechanism was elucidated through stoichiometric experiments, initiating the catalytic cycle from the active calcium hydride species. Subsequently, the [Ca]–H species reacts with pyridine to yield the [Ca]-1,2-DHP complex (**Int 18**, Figure 9), which then reacts with silanes to form an ion pair containing hypervalent silicon species (Intemann et al., 2015). Ultimately, hydride transfer to the cationic calcium species occurs, leading to the release of the *N*-silyl enamine product and regeneration of the active [Ca]–H complex.

### 3.2 Borane-catalyzed 1,4-hydrosilylation

Chang et al. recently reported a B(C_6_F_5_)_3_-catalyzed hydrosilylation with a broad substrate scope that encompasses the dearomatization of *N*-heteroarenes, such as quinoline, isoquinoline, and pyridine, as well as the reduction of conjugated nitriles and imines.

In their catalytic system, the dearomatization of *N*-heteroarenes proceeded via the formation of *N*-silyl enamine intermediates prior to yielding 1,3-bis-silylated products (Gandhamsetty et al., 2014; Gandhamsetty et al., 2015a). Moreover, the selectivity of *N*-silyl enamine formation in the initial step was different for each *N*-heteroarene. Quinolines and 2- and 3-substituted pyridines underwent 1,4-hydrosilylation selectively. Isoquinolines and 4-substituted pyridines underwent 1,2-hydrosilylation because of the effects of aromaticity and steric hindrance (Gandhamsetty et al., 2014; Gandhamsetty et al., 2015b). Additionally, the formation of linear *N*-silyl enamine through 1,4-addition was also observed when utilizing α,β-unsaturated nitriles and α,β-unsaturated aldimines (Gandhamsetty et al., 2015a; Kim et al., 2018). Kinetic and control experiments demonstrated the accumulation of *N*-silyl enamine intermediates until complete consumption of *N*-heteroarenes occurred. The conversion to the fully reduced products was initiated at the peak *N*-silyl enamine concentrations (Gandhamsetty et al., 2014; Gandhamsetty et al., 2015a; Gandhamsetty et al., 2015b; Kim et al., 2018). This finding proves that precise control of the amount of silane can achieve exclusive *N*-silyl enamine formation. *N*-silyl-1,4-dihydropyridines were synthesized using 1.1 equivalent of Me_2_PhSiH (1.1 equiv.) and the synthesis of linear *N*,*N*-disilyl enamines could be achieved by adjusting the stoichiometry of bulky silanes in Chang’s system (Gandhamsetty et al., 2015b). The utilization of equimolar quantities of HSiMe_2_Ph resulted in *N*-silyl-1,4-dihydroquinolines and *N*-silyl-1,2-isoquinolines (Petrushko and Nikonov, 2020).

**FIGURE 10:**
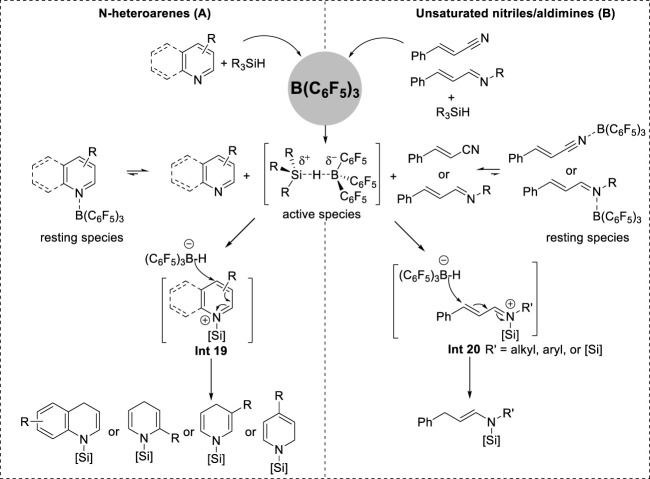
**(A)** Borane-catalyzed 1,4-hydrosilylation of *N*-heteroarenes. **(B)** Borane-catalyzed 1,4-hydrosilylation of unsaturated nitriles/aldimines.

Based on experimental studies and density functional theory (DFT) calculations of the reaction mechanism, the formation of *N*-silyl enamine intermediates in borane-catalyzed hydrosilylation was concluded to occur via an ionic mechanism (Figure 8) (Gandhamsetty et al., 2014; Gandhamsetty et al., 2015a; Gandhamsetty et al., 2015b; Kim et al., 2018). Initially, B(C_6_F_5_)_3_ coordinates to the N center to establish a stable resting species. These species exist in equilibrium with both the free reactants and the active complex (C_6_F_5_)_3_B∙HSiR_3_. This active complex facilitates the transfer of its silylium cation to the reactants, leading to the formation of iminium salt **Int 19** (Figure 10) or **Int 20** (Figure 10) along with the borohydride anion [(C_6_F_5_)_3_BH]^−^. Subsequently, the borohydride anion transfers the hydride to the C_4_ site of **Int 19** or **Int 20**, forming cyclic *N*-silyl enamines.

### 3.3 Transition-metal-catalyzed hydrosilylation

**FIGURE 11:**
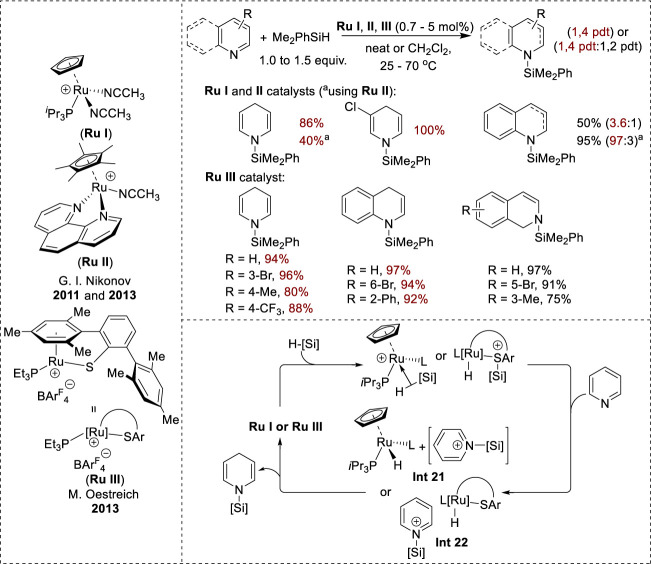
Transition-metal-catalyzed 1,4-hydrosilylation of *N*-heteroarenes.

Because of their similar ionic mechanisms, various cationic Ru complexes, including [Cp(^*i*^Pr_3_P)Ru(NCCH_3_)_2_]^+^ (**Ru I**), [Cp(phen)Ru(NCCH_3_)_2_]^+^ (**Ru II**), and coordinatively unsaturated Ru^II^ thiolate (**Ru III**), exhibit 1,4-selectivity in the hydrosilylation of *N*-heteroarenes (Figure 11). **Ru I** and **Ru II** display good conversions with 3- and 5-substituted pyridines, whereas 2-, 4-, and 6-substituents were ineffective (Gutsulyak et al., 2011; Lee et al., 2013). Conversely, **Ru III** reduces various *N*-heteroarenes, including pyridines, isoquinolines, and quinolines, resulting in high regioselectivity and chemoselectivity. Notably, 4-substituted pyridines react effectively to provide high yields of *N*-silyl-1,4-dihydropyridines (Königs et al., 2013). The proposed mechanism for the formation of **Ru I** and **Ru II** complexes begins with the formation of cationic silane complexes from Ru via nitrile dissociation and silane coordination. Cationic silane complexes facilitate the transfer of a silyl cation to the pyridine substrate to form **Int 21** (Figure 11) and a reactive Ru hydride species, which then induces hydride transfer to the 4-position to yield the *N*-silyl enamine product and regenerate the active catalyst complex (Gutsulyak et al., 2011; Lee et al., 2013). In addition, the mechanism for the **Ru III** complex starts with the formation of a cationic silicon–sulfur intermediate via the activation of silane by the heterolytic cleavage of the Si–H bond. Subsequently, the cationic silicon–sulfur intermediate transferred the cationic silicon to the nitrogen center of pyridine, forming the *N*-silylpyridinium intermediate (**Int 22**, Figure 11) and neutral Ru hydride. Eventually, the neutral Ru hydride attacked the C4-position in the *N*-silyl pyridinium intermediate to generate the *N*-silyl enamine products (Bähr and Oestreich, 2018).

**FIGURE 12:**
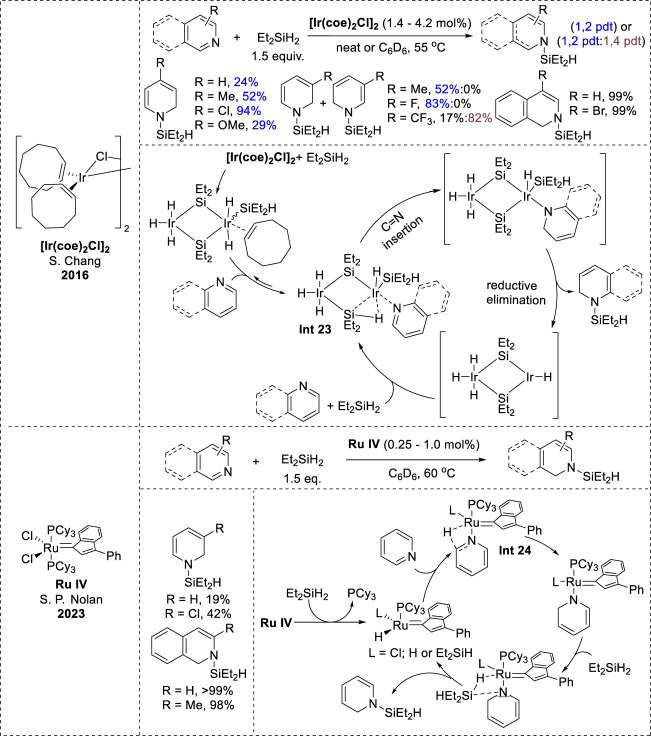
Transition-metal-catalyzed 1,2-hydrosilylation of *N*-heteroarenes.

In addition to 1,4-hydrosilylation, transition-metal-catalyzed 1,2-hydrosilylation was demonstrated using a metathesis-active ruthenium complex (**Ru IV**) and an iridium catalyst ([Ir (coe)_2_Cl]_2_). These catalysts were versatile, functioned effectively to *N*-heteroarenes, and displayed excellent tolerance to various functional groups (Jeong et al., 2016; Ma and Nolan, 2023). The mechanisms of both the catalytic systems follow an inner-sphere path. In the iridium complex mechanism, the initial step involves the generation of two isomeric iridium olefin adducts via a reaction between [Ir (coe)_2_Cl]_2_ and Et_2_SiH_2_. These adducts undergo ligand exchange with *N*-heteroarenes to form the bimetallic species (**Int 23,** Figure 12), which undergoes intramolecular insertion of an Ir–H bond into the C=N bond of the *N*-heteroarene ligand, generating a 1,2-dihydropyridine intermediate. Subsequently, the 1,2-dihydropyridine intermediate produces the *N*-silyl enamine through reductive elimination (Jeong et al., 2016). In the Ru complex mechanism, the Ru complex transforms into the activated species via PCy_3_ dissociation and ligand exchange. The *N*-heteroarene coordinates with the ruthenium species (**Int 24**, Figure 12), forming a 1,2-reduced intermediate via selective hydride transfer at the 2-position. Eventually, the 1,2-reduced intermediate undergoes σ-bond metathesis with another silane molecule, resulting in the release of *N*-silyl enamine (Ma and Nolan, 2023).

## 4 Transition-metal catalysis of both hydroboration and hydrosilylation

**FIGURE 13:**
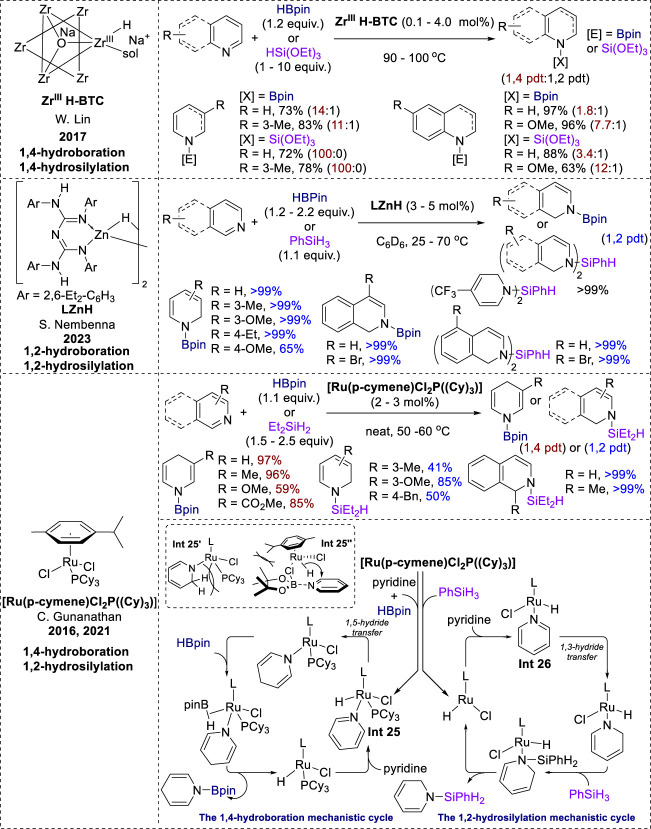
Transition-metal catalysis of both hydroboration and hydrosilylation.

In 2017, Lin et al. reported a zirconium framework, Zr^III^H-BTC, that exhibited high activity and 1,4-selectivity in the dearomatization of pyridines and quinolines using HBpin and triethoxysilane (Figure 13). This selectivity was attributed to the bridging oxo/carboxylate ligands and the site-isolation effect of the MOF, which stabilized the coordinatively unsaturated Zr^III^H centers (Ji et al., 2017). Moreover, 1,2-selective hydroboration and hydrosilylation of *N*-heteroarenes were achieved under β-diketiminate-supported dimeric zinc hydride [LZnH]_2_ conditions by Nembena in 2023 (Sahoo et al., 2023). A large range of 3- and 4-substituted pyridines were transformed into the 1,2-hydroborated products in excellent yields. Similar to other 1,2-selective systems, 2-substituted pyridines failed to produce the reductive products. Interestingly, the hydrosilylation of pyridines and isoquinolines in this catalytic system produced bis-hydrosilylated products in quantitative yield. Similar to other metal-hydride-catalyzed hydroboration and hydrosilylation reactions, the reaction mechanism involves a 1,3-hydride transfer to furnish zinc amide intermediates. Further addition of HBpin or a hydrosilane to the amide intermediates produced selective 1,2-hydroborylated and hydrosilylated products.

Additionally, the Ru (II) precatalyst [Ru (p-cymene)Cl_2_(P(Cy)_3_)] exhibited distinct selectivities for both 1,4-hydroboration and 1,2-hydrosilylation in Gunanathan’s research. The hydroboration of various 3-substituted pyridines in this catalytic system took place with efficient 1,4-selectivity, with good-to-excellent yields. However, 2-substituted and 4-substituted pyridines showed no reactivity towards the ruthenium-catalyzed 1,4-hydroboration (Kaithal et al., 2016). Additionally, this catalyst facilitated the 1,2-hydrosilylation of pyridines, affording moderate product yields for the meta- and para-substituted derivatives. Isoquinoline also underwent efficient conversion, affording *N*-silyl-1,2-dihydroisoquinoline in quantitative yield (Behera et al., 2021). The formation of the **Int 25** (Figure 13) and **Int 26** (Figure 13) species determined the distinct selectivity between the two processes. In the hydroboration mechanism, **Int 25** is formed by the reaction of a ruthenium precatalyst with HBpin and pyridines in the presence of a phosphine ligand (PCy_3_) (Kaithal et al., 2016). However, during hydrosilylation, the Ru precatalyst dissociates from the PCy_3_ ligand before reacting with silane and pyridine to form **Int 26**. Subsequently, **Int 26** undergoes 1,3-hydride transfer to form a dihydropyridine intermediate, whereas **Int 25** prefers 1,5-hydride transfer. The different selectivity between 1,2-hydrosilylation and 1,4-hydroboration under the same ruthenium precatalyst is initially attributed to the steric hindrance between the “sp^3^-CH_2_” of the 1,2-dihydropyridine ligand and the phosphine ligand (**Int 25**′, Figure 13) (Kaithal et al., 2016). Additionally, they also performed kinetic studies on this hydroboration to support the intramolecular 1,5-hydride shift mechanism. However, subsequent DFT calculations revealed that the 1,2-hydroboration process was impeded due to steric effects between the methyl groups of HBpin and the *p*-cymene ligand in a ruthenium catalyst (**Int 25″**, Figure 13), thereby promoting 1,4-selectivity in hydroboration (Behera et al., 2021). Eventually, the 1,4- or 1,2-dihydropyridine intermediates undergoes subsequent metathesis with HBPin or silane, leading to the formation of the *N*-boryl or *N*-silyl enamine products.

## 5 Applications of *N*-boryl and *N*-silyl enamines in organic synthesis

**FIGURE 14:**
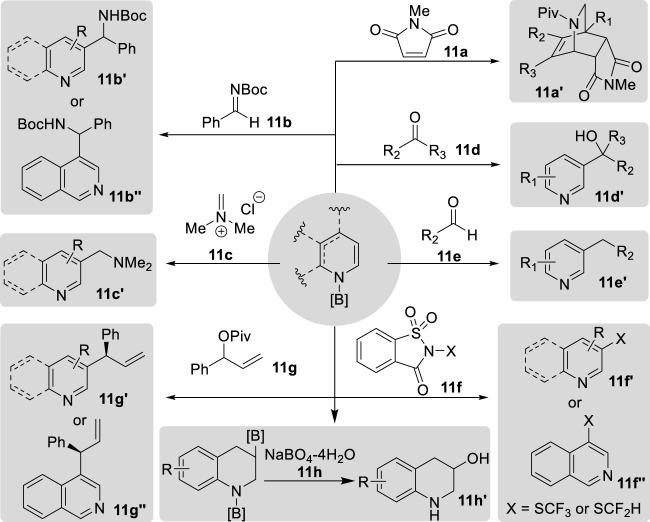
Applications of N-boryl enamines.

*N*-Boryl and *N*-silyl enamines serve as versatile nucleophilic motifs that can react with a broad range of electrophilic reagents. Recent synthetic methodologies have predominantly focused on the functionalization of the C_3_-position of borylated and silylated enamines through nucleophilic attack on external electrophiles, as well as the facilitation of the construction of complex molecular scaffolds via cycloaddition reactions involving the C=C moiety and various dipoles (Figure 14). Remarkably, the *in situ* utilization of *N*-boryl and *N*-silyl enamines enables efficient one-pot tandem reactions. *N*-boryl and *N*-silyl enamines have similar reactivities; the choice of one over the other is dictated by the specific cleavage pathways of their respective silyl and boryl groups in the final product, which may involve rearomatization, carbonylation, or facile N–H bond formation under acidic conditions.

The application of borylated enamines in Diels–Alder reactions was described by Suginome et al., who synthesized *N*-boryl-1,2-dihydropyridines synthesized from pyridines using [RhCl(cod)]_2_/PCy_3_ as a catalyst. This *N*-boryl enamine reacts with *N*-methyl maleimide **11a** via [4 + 2] cycloaddition, to yield isoquinuclidine derivatives **11a′**. Subsequent acylation of the B–N bond with pivaloyl chloride afforded the final products in good yields (Oshima et al., 2012). Moreover, Wang et al. reported the C3-selective functionalization of pyridine through a tandem process involving the reaction of *N*-boryl-1,4-dihydropyridines synthesized via the borane-catalyzed hydroboration of pyridines with various electrophilic reagents, ultimately leading to the desired products via oxidative aromatization of the adducts in air without the need for additional oxidants (Liu et al., 2022; Zhou et al., 2022). When the imine **11b**, iminium **11c**, and carbonyl compounds **11d**–**11e** were employed as electrophiles, exclusive regioselectivity for C3 carbon–carbon bond formation in **11b′–11e′** or C3 carbon–sulfur bond formation in **11f′** and **11f″** was observed (Liu et al., 2022; Zhou et al., 2022). Specifically, the treatment of 3-substituted pyridine under these conditions yielded moderate-to-high yields of monofunctional derivatives, whereas 4-substituted and 2-substituted pyridines yielded difunctional derivatives at the 3- and 5-positions. Notably, ketones yielded the alcohol products **11e′**, whereas aldehydes afforded the dehydroxylated products **11f′** (Liu et al., 2022). Recently, building upon this tandem procedure, the authors successfully synthesized the C3-allylated pyridines **11g′** and **11g″** with allylic ester **11g** via enantioselective iridium-catalyzed allylation (Liu et al., 2023). Additionally, the excess HBpin also acted as an electrophilic reagent, which reacts with *N*-boryl 1,4-dihydroquinolines to produce C3-borylated tetrahydroquinolines in the borane-catalyzed double hydroboration of quinolines. Chang’s group synthesized various 3-hydroxytetrahydroquinolines **11h′** after oxidation of 1,3-*bis*-borylated tetrahydroquinolines by sodium borate **11h** (Kim et al., 2020).

**FIGURE 15:**
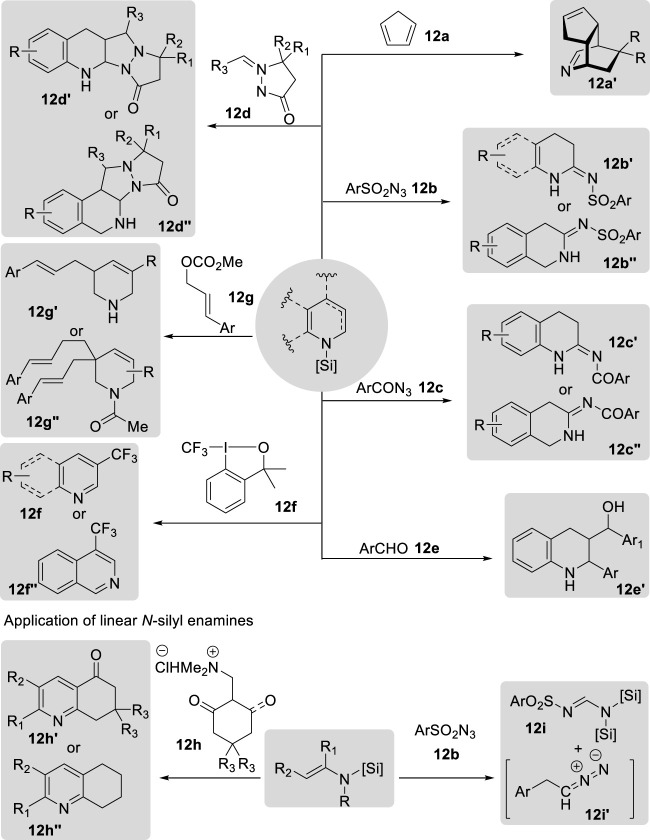
Application of *N-*silyl enamines.

Cyclic *N*-silyl enamines have been utilized to synthesize polycyclic structures via Diels–Alder reactions (Figure 15). Wanner et al. pioneered the [4 + 2] cycloaddition of *N*-silyl-1,4-dihydropyridine with cyclopentadiene **12a** to synthesize 2-azabicyclo [2.2.2]octane (Schmaunz et al., 2014). The *N*-silyl-1,4-dihydropyridine precursor was prepared by the reaction of pyridines with triisopropylsilyl triflate, yielding the corresponding pyridinium salts, which were subsequently trapped in diorganomagnesium compounds. After [4 + 2] cycloaddition, 5,5-diarylsubstituted 2-azabicyclo [2.2.2]octane derivatives **12a′** were obtained in good-to-high yields. These derivatives underwent intramolecular electrophilic aromatic substitution reactions to produce 7,8-benzomorphane derivatives upon treatment with acetyl chloride. In contrast, subsequent studies by Joung’s group focused on the [3 + 2] cycloaddition of cyclic *N-*silyl enamines via the borane-catalyzed hydrosilylation of *N-*heteroarenes with organic azides **12b, 12c** or azomethine imines **12d** (Cao et al., 2020a; Cao et al., 2020b; Cao et al., 2022; Jo et al., 2022). Borane-catalyzed hydrosilylations yield a broad range of cyclic *N-*silyl enamines from various *N-*heteroarenes. These enamines could be utilized *in situ* without further purification, thereby facilitating the development of a one-pot procedure. After the [3 + 2] cycloaddition of *N-*silyl enamines with organic azides, including sulfonyl azide **12b** and carbonyl azide **12c**, amidines **12b′**, **12b″** and **12c′**, **12c″** were produced in good-to-high yields. The formation of amidines occurs through the reaction of *N-*silyl enamines and azides, generating a triazoline intermediate that immediately rearranges to a cyclic amidine via hydride shift and nitrogen gas extrusion (Cao et al., 2020a; Cao et al., 2020b; Jo et al., 2022). In addition, the authors discovered that these *N-*silyl enamines also underwent [3 + 2] cycloaddition with azomethine imine **12d**, resulting in the formation of tetracyclic pyrazolidinone scaffold structures **12d′** in good yield. The [3 + 2] cycloaddition process encompasses both endo and exo pathways (Cao et al., 2022).

In addition to their application in cycloaddition reactions, cyclic *N-*silyl enamines serve as nucleophilic motifs for various chemical transformations. Crudden et al. reported the synthesis of γ-aminoalcohols **12e′** derived from quinoline via dearomatization under borenium-catalyzed hydrosilylation, resulting in the formation of *N-*silyl-1,4-dihydroquinolines (Clarke et al., 2021). These *N-*silyl-1,4-dihydroquinolines were then added to aldehyde **12e**, followed by reduction using NaBH_4_ and subsequent deprotection of the silyl group to yield tetrahydroquinoline derivatives in modest-to-good yields. Furthermore, these *N-*silyl enamines derived from the borane-catalyzed hydrosilylation of *N-*heteroarenes have also been employed for the production of 3-trifluoromethylated compounds **12f′** and **12f′′** via electrophilic trifluoromethylation with Togni reagent I (**12f**) (Muta et al., 2022). Remarkably, research conducted by Stoltz et al. utilized *N-*silyl enamines derived from iridium(I)-catalyzed dearomative 1,2-hydrosilylation as nucleophiles in a subsequent palladium-catalyzed asymmetric allylic alkylation with cinnamyl methyl carbonate **12g**, leading to the formation of C3-substituted tetrahydropyridine products **12g′** and **12g′′** (Greßies et al., 2023). The desired products were isolated in moderate yields with excellent enantioselectivity following carbonylation with acetyl chloride. Moreover, bisalkylated tetrahydropyridines **12g′′** were obtained in the presence of benzoic acid and an excess of the allyl carbonate substrate. The formation of the bisalkylated products arose from the tautomerization of the imine intermediate, resulting in an enamine that could participate in additional alkylation.

Similarly, Sakai et al. developed a Yb(OTf)_3_-catalyzed cyclization process utilizing the nucleophilicity of linear *N-*silyl enamines to synthesize quinolinone and tetrahydroquinoline derivatives from endione and enone precursors **12h** (Sakai et al., 2006). This process yielded the desired cyclic products **12h′** and **12h′′** in good yields; the quinolinone derivatives were further transformed into substituted quinolines upon treatment with NBS, AIBN, and *p*-toluenesulfonic acid in methanol. Joung et al. reported the use of linear *N-*silyl enamines in [3 + 2] cycloaddition reactions with sulfonyl azides **12b** (Do et al., 2024). The authors synthesized linear *N-*silyl enamines through borane-catalyzed hydrosilylation of α,β-unsaturated nitriles. However, unlike the rearrangement of a triazoline intermediate from cyclic *N-*silyl enamines and azides, linear *N-*silyl enamines reacted with sulfonyl azides to generate a triazoline intermediate, which underwent a distinct retro-(3 + 2) cycloaddition to produce the corresponding formamidine **12i′** and versatile alkyl diazomethane **12i′′**.

## 6 Summary

This review described the formation of *N-*boryl and *N-*silyl enamines through the hydroboration and hydrosilylation of a range of conjugated systems employing a variety of catalysts. The hydrides for the reduction process originate from *in situ* formed catalysts, the dissociation of B–H or Si–H bonds, or coordination of HBPin or silane as ligands of the catalyst. The selectivity between 1,2- and 1,4-addition arises from the formation of distinct intermediates. Specifically, active metal hydrides induce an intramolecular 1,3-hydride shift in the metal-substrate complex, leading to the formation of 1,2-adducts. Bulky active hydrides or sterically hindered ligands promote a hydride shift to the less-hindered C4-position. Isomerization from kinetic 1,2-adducts to thermodynamically stable 1,4-adducts can also afford 1,4-adducts. As an application of these *N-*boryl and *N-*silyl enamines, the utilization of the various functional *N-*heterocyclic structures obtained from the diverse regioselective reductions of *N*-boryl and *N-*silyl enamines described above facilitates the development of one-pot or tandem procedures for subsequent chemical transformations of the versatile *N-*boryl and *N-*silyl enamines.
